# “What are the effects of diagnostic imaging on clinical outcomes in patients with low back pain presenting for chiropractic care? A matched observational study.” Jenkins et al., Chiropractic & Manual Therapies 2021;29:46

**DOI:** 10.1186/s12998-022-00420-w

**Published:** 2022-03-01

**Authors:** Mark A. Lopes

**Affiliations:** Gonstead Clinical Studies Society, 2107 Forest Ave, Suite 130, Chico, CA 95928 USA

Dear Editor,

Studies such as the one that is the subject of this letter to the editor [1] are important, and I commend the authors for their efforts. Their conclusions were that diagnostic imaging did not result in better outcomes. However, as described in the article, the findings from the paper support a more limited conclusion, such as "imaging did not appear to improve outcomes when used in an unspecified manner." We are left to make assumptions regarding how imaging was used to influence treatment, if at all.

The primary purpose of imaging, as described, was apparently to rule out red flags, which is also supported by the 24% referral rate for imaging. Imaging alone cannot be expected to produce different outcomes between those that received imaging and those that didn't.

This study did not control or describe treatment specifically or address how and to what extent imaging was used to inform treatment decisions. The patients in both the imaging group and the non-imaging group may or may not have been treated similarly. We cannot, therefore, suggest from this study that the lack of difference in outcomes between groups was due to the lack of additional benefits of treatment informed by imaging.

I hope others continue such studies, but there is a need to describe how imaging was used to inform treatment in order to determine whether or not imaging is useful for biomechanical or other assessments prior to treatment if imaging is to be used beyond red-flag purposes.

## Data Availability

Not applicable.
